# Association between red blood cell distribution width-to-albumin ratio and in-hospital mortality in patients with congestive heart failure combined with chronic kidney disease

**DOI:** 10.3389/fcvm.2025.1563512

**Published:** 2025-07-04

**Authors:** Lie-jun Qian, Lu-xi Hong

**Affiliations:** ^1^ ICU, Tiantai County Hospital of Traditional Chinese Medicine, Taizhou, Zhejiang, China; ^2^ Department of Ultrasound, Tiantai County People’s Hospital, Taizhou, Zhejiang, China

**Keywords:** congestive heart failure combined with chronic kidney disease, blood cell distribution width-to-albumin ratio, in-hospital mortality, machine learning algorithms, mediation analysis

## Abstract

**Background:**

This study aimed to explore the relationship between the blood cell distribution width-to-albumin ratio (RAR) and in-hospital mortality in patients with congestive heart failure (CHF) combined with chronic kidney disease (CKD).

**Methods:**

The patients' information was collected from the Medical Information Mart for Intensive Care IV (MIMIC-IV) database. The association of the RAR and in-hospital mortality were assessed via logistic regression analysis with adjusting for different covariate, followed by visualization via the restricted cubic splines (RCS) analysis. Correlation analysis and machine learning algorithms were used to screen the clinical features associated with RAR. Mediation analysis was used to explore the function of RAR-related feature on their association. The receiver operating characteristic (ROC) curve and decision curve analysis (DCA) curves were used to explore the efficacy of RAR in predicting patient outcomes and the net clinical benefit, respectively.

**Results:**

A total of 1,243 patients were included in this study. Logistic regression analysis showed that RAR was a risk factor of in-hospital death. RCS showed a linear relationship between RAR and in-hospital mortality, which was not affected by confounding factors. Monocytes played a mediating role in the relationship between RAR and in-hospital mortality. RAR had a good efficacy and clinical net for predicting in-hospital mortality, half-year mortality, one-year mortality, and three-year mortality.

**Conclusions:**

We found that RAR was a risk factor for in-hospital death and could predict short-term and long-term outcomes in patients with CHF combined with CKD. Monocytes played a mediating role in their relationship.

## 1 Introduction

Congestive heart failure (CHF) is a complex clinical syndrome characterized by impaired cardiac function, leading to systemic congestion and multi-organ dysfunction. Among the comorbidities associated with CHF, renal dysfunction is particularly prevalent and up to 50% of patients with CHF exhibit some degree of renal impairment (1). The coexistence of CHF combined with chronic kidney disease (CKD), often referred to as cardiorenal syndrome, is associated with worse clinical outcomes, including increased morbidity and mortality (2). Specifically, patients with CHF and concurrent renal dysfunction are at a significantly higher risk of in-hospital mortality compared to those with CHF alone (3).

The pathophysiology linking CHF combined with CKD involves a vicious cycle of hemodynamic and neurohormonal alterations. Reduced cardiac output in CHF leads to decreased renal perfusion, activating the renin-angiotensin-aldosterone system (RAAS) and sympathetic nervous system, which further exacerbates fluid retention and cardiac workload (4). This interplay not only accelerates the progression of both cardiac and renal dysfunction but also complicates the management of these patients, making early risk stratification crucial.

In recent years, there has been growing interest in identifying biomarkers that can predict adverse outcomes in patients with CHF combined with kidney disease. Among those patients, the red blood cell distribution width (RDW) has recently been proven to be an effective marker for determining the prognosis of heart failure (5, 6). RDW, a measure of variability in red blood cell size, was associated with increased mortality in various cardiovascular conditions, including CHF (7). Elevated RDW levels are thought to reflect underlying inflammation, oxidative stress, and nutritional deficiencies, all of which are prevalent in CHF patients (8).

Albumin, a marker of nutritional status and systemic inflammation, was also independently linked to outcomes in different diseases. Hypoalbuminemia is common in patients with atrial fibrillation and is associated with increased mortality (9). The combination of RDW and albumin into a single metric, the RDW-to-albumin ratio (RAR), has recently been proposed as a novel prognostic tool. Preliminary studies suggest that RAR may provide superior predictive value for adverse outcomes compared to either marker alone, particularly in high-risk populations with cardiovascular disease (10–12), sepsis (13), and aortic aneurysms (14).

Given the high in-hospital mortality risk in patients with CHF combined with CKD, there is a critical need for reliable and easily accessible biomarkers to aid in risk stratification. The RAR, which integrates information on both inflammation and nutritional status, holds promise as a predictor of in-hospital mortality in this vulnerable population. This study aims to retrospectively evaluate the prognostic value of RAR in predicting in-hospital mortality among patients with CHF and concurrent renal dysfunction, with the hypothesis that elevated RAR levels are associated with increased mortality risk.

## 2 Materials and methods

### 2.1 Data source and study population

All data for this study were obtained from the Medical Information Mart for Intensive Care IV(MIMIC-IV) database. The database is an open public database, approved by the institutional review boards of Beth Israel Deaconess Medical Center and Massachusetts Institute of Technology, and collects information on patients hospitalized in the intensive care unit (ICU) from 2008–2019. In addition, the database was anonymized to protect patient privacy, thus informed consent was waived. The personnel who extracted the data for this study had passed the training program exam (CITI program) and received a license to use the MIMIC-IV database. PostgreSQL software was used to extract the data.

Inclusion criteria: (1) patients who were eligible for CHF with CKD, (2) age greater than or equal to 18 years. Exclusion criteria: (1) patients with missing albumin and RDW; (2) no record of in-hospital mortality, half-year, one-year, and three-year mortality follow-up. Based on the inclusion criteria, we collected 5,153 patients' data, excluded 2,790 cases with missing data RAR, and excluded 917 cases without follow-up data. Finally, 1,243 cases were included in the study. Notably, both CHF and CKD were identified exclusively through the International Classification of Diseases (ICD) code (Supplementary document 1).

### 2.2 Variables and outcomes

In this study, we collected basic demographic data of the patients: gender (male, female), race (white, non-white), marital status (single, married, divorced, widowed); hospitalization record: length of hospitalization, laboratory markers: albumin (g/dl), alanine aminotransferase (ALT, IU/L), anion gap (mEq/L), aspartate aminotransferase (AST, IU/L), bicarbonate (mEq/L), blood urea nitrogen (mg/dl), calcium (mg/dl), chloride (mEq/L), creatinine (mg/dl), glucose (mg/dl), hematocrit (%), hemoglobin (g/dl), international normalized ratio (INR), platelets (×10^9^/L), potassium (mEq/L), prothrombin time (PT, sec), red blood cell (RBC, m/ul), RDW, (%), sodium, (mEq/L), total bilirubin, (mg/dl), white blood cell (WBC, K/ul), creatine phosphorylation kinase (CPK, IU/L), creatine kinase isoenzyme B (CKMB, ng/ml), urea nitrogen (mg/dl), monocyte (%), estimated glomerular filtration rate (EGFR, ml/min), RDW-to-albumin ratio (RAR); comorbidities: infarction, hypertension, stroke, diabetes mellitus(DM), atrial fibrillation (AF); and medication record: β-receptor blocker (Metoprolol, Atenolol, Carvedilol) using, angiotensin-converting enzyme inhibitor/angiotensin receptor blocker (ACE-I/ARB) using (Captopril, Enalapril, Benazepril, Valsartan, Losartan), calcium inhibitor (Nifedipine, Amlodipine) using, diuretic (Furosemide, Hydrochlorothiazide, Acetazolamide) using. Patients were classified as β-receptor blocker users if they received any agent within a given drug class (Metoprolol, Atenolol, Carvedilol). Similarly, users of the other three types of drugs were defined in this way. However, extensive missing dosage data precluded quantification of dose ranges and mean doses for non-adherent individuals. All of the above variables were covariates, which were all collected from the first hospitalization.

We calculated the RAR of the patients according to the following formula: RAR = RDW (%)/albumin (g/dl). RAR was the independent variable of this study. In-hospital mortality was the study's primary outcome, with half-year mortality, one-year mortality, and three-year mortality as secondary outcomes. The date of death was extracted from two sources: the hospital information system and the Massachusetts State Registry of Vital Records and Statistics. Individual patient records from MIMIC were matched to the vital records using a custom algorithm based on identifiers including name, social security number, and date of birth.

### 2.3 Statistical analysis

Patients were divided into 2 groups according to in-hospital mortality status, and continuous variables that conformed to normal distribution between the two groups were compared using the *t*-test and expressed as mean and standard deviation, while data that did not conform to normal distribution were compared using the *U*-test and expressed as median and quartiles. Categorical variables between the two groups were compared using the chi-square test and expressed as frequencies and percentages. Logistic regression analysis was used to analyze the relationship between RAR and in-hospital mortality adjusting for different laboratory index or clinical features between the alive group and the death group (model 1 adjusted for ALT, anion gap, AST, bicarbonate, calcium, glucose, INR, platelets, total bilirubin WBC, CPK, monocyte, EGFR; model 2 adjusted for sex, calcium inhibitor, race, smoking, stroke, AF) and their association was visualized using restricted cubic spline (RCS). The receiver operating characteristic (ROC) curve and decision curve analysis (DCA) curves were used to explore the efficacy of RAR in predicting patient outcomes and the net clinical benefit, respectively. Correlation analysis and machine learning algorithms were used to screen for clinical features most relevant to RAR. Mediation analysis was used to explore the mediating role of the clinical characteristics most relevant to RAR on the relationship between RAR and the in-hospital death. All analyses were performed using R software (version 4.2.2), with *P* < 0.05 as the criterion for a significant difference.

## 3 Results

### 3.1 Baseline characteristics of the participants

A total of 1,243 patients, comprising 1,023 survivors and 220 deaths were included. The patients' age ranged from 25–91 years, with a median age of 78. The proportion of males was 58.57%, and the proportion of females was 41.43%. There was a statistically significant difference in ALT, anion gap, AST, bicarbonate, calcium, glucose, INR, platelets, total bilirubin WBC, CPK, monocyte, EGFR, RDW, albumin, RAR, sex, calcium inhibitor, race, smoking, stroke, and AF between the survival and death groups (Tables 1, 2, *P* < 0.05). It is noteworthy that the RAR of the death group was significantly higher than that of the survival group (5.387 vs. 4.788) (Table 1, *P* < 0.05).

**Table 1:** Clinical characteristics (continuous variable) of patients.

Variables	Alive (*n* = 1,023)	Death (*n* = 220)	z	*P*
Age, (years)	78.000 (68.000, 85.000)	79.000 (71.000, 84.000)	−0.241	0.810
Length of stay, (day)	7.895 (4.678, 13.099)	8.888 (4.960, 15.819)	−1.819	0.069
Albumin, (g/dl)	3.300 (2.900, 3.700)	3.000 (2.500, 3.500)	5.499	<0.001
ALT, (IU/L)	20.000 (14.000, 36.000)	25.000 (14.000, 48.000)	−2.702	0.007
Anion gap, (mEq/L)	16.000 (13.000, 18.000)	16.000 (14.000, 19.000)	−3.009	0.003
AST, (IU/L)	28.000 (19.000, 45.000)	39.000 (23.000, 76.000)	−4.935	<0.001
Bicarbonate, (mEq/L)	24.000 (21.000, 28.000)	23.000 (20.000, 26.000)	3.364	<0.001
Blood urea nitrogen, (mg/dl)	40.000 (29.000, 58.000)	44.000 (29.000, 68.000)	−1.847	0.065
Calcium, (mg/dl)	8.700 (8.100, 9.100)	8.600 (8.000, 9.000)	2.510	0.012
Chloride, (mEq/L)	102.000 (98.000, 106.000)	101.000 (97.000, 107.000)	1.069	0.284
Creatinine, (mg/dl)	2.000 (1.400, 3.100)	2.000 (1.400, 2.900)	0.304	0.761
Glucose, (mg/dl)	120.000 (96.000, 159.000)	129.000 (102.000, 186.000)	−2.649	0.008
Hematocrit, (%)	31.800 (28.200, 35.700)	31.300 (28.000, 35.500)	0.296	0.768
hemoglobin, (g/dl)	10.300 (9.100, 11.600)	10.200 (9.000, 11.500)	0.670	0.503
INR	1.300 (1.100, 1.700)	1.400 (1.200,1.800)	−2.307	0.020
Platelets, (×10^9^/L)	203.000 (151.000, 264.000)	186.000 (129.000, 256.000)	2.276	0.023
Potassium, (mEq/L)	4.300 (3.900, 4.800)	4.300 (3.900, 4.800)	−0.827	0.408
PT, (sec)	14.400 (12.700, 18.900)	14.900 (13.000, 20.100)	−1.814	0.070
RBC, (m/ul)	3.450 (3.060, 3.920)	3.340 (3.030, 3.900)	0.953	0.340
RDW, (%)	15.500 (14.400, 17.100)	16.200 (14.600, 17.600)	−2.729	0.006
Sodium, (mEq/L)	139.000 (136.000, 141.000)	138.000 (134.000, 141.000)	1.923	0.054
Total bilirubin, (mg/dl)	0.500 (0.300, 0.900)	0.600 (0.400, 1.400)	−3.989	<0.001
WBC, (K/ul)	8.400 (6.300, 11.600)	10.000 (7.400, 14.700)	−4.630	<0.001
CPK, (IU/L)	75.000 (42.000, 153.000)	97.000 (43.000, 240.000)	−2.118	0.034
CKMB, (ng/ml)	91.000 (69.000, 132.000)	99.000 (72.000, 138.000)	−1.542	0.123
Urea nitrogen, (mg/dl)	40.000 (29.000, 58.000)	44.000 (29.000, 68.000)	−1.847	0.065
Monocyte, (%)	4.300 (2.700, 6.000)	3.000 (1.800, 5.000)	5.091	<0.001
EGFR, (ml/min)	0.995 (0.788, 1.025)	1.005 (0.796, 1.027)	−2.178	0.029
RAR	4.778(4.184, 5.727)	5.387(4.622, 6.577)	−6.123	<0.001

ALT, alanine aminotransferase; AST, aspartate aminotransferase; INR, international normalized ratio; PT, prothrombin time; RBC, red blood cell; RDW, red blood cell distribution width; WBC, white blood cell; CPK, creatine phosphorylation kinase; CKMB, creatine kinase isoenzyme B; EGFR, estimated glomerular filtration rate; RAR, red blood cell distribution width-to-albumin ratio.

**Table 2:** Clinical characteristics (categorical variables) of patients.

Variables	Alive (*n* = 1,023)	Death (*n* = 220)	*χ* ^2^	*P*
Race, *n* (%)				
White	740 (76.446)	142 (84.024)	4.748	0.029
No-white	228 (23.554)	27 (15.976)		
Marital status, *n* (%)				
Single	183 (18.392)	37 (18.137)	0.533	0.912
Married	470 (47.236)	97 (47.549)		
Divorced	79 (7.940)	19 (9.314)		
Widowed	263 (26.432)	51 (25.000)		
Sex, *n* (%)				
Male	582 (56.891)	146 (66.364)	6.695	0.010
Female	441 (43.109)	74 (33.636)		
Alcohol use, *n* (%)				
No	955 (93.353)	199 (90.455)	2.288	0.13
Yes	68 (6.647)	21 (9.545)		
Smoking, *n* (%)				
No	648 (63.343)	168 (76.364)	13.612	<0.001
Yes	375 (36.657)	52 (23.636)		
MI, *n* (%)				
No	832 (81.329)	165 (75.000)	4.569	0.033
Yes	191 (18.671)	55 (25.000)		
DM, *n* (%)				
No	506 (49.462)	109 (49.545)	0.001	0.982
Yes	517 (50.538)	111 (50.455)		
Stroke, *n* (%)				
No	905 (88.465)	106 (48.182)	193.546	<0.001
Yes	118 (11.535)	114 (51.818)		
Hypertension, *n* (%)				
No	952 (93.060)	197 (89.545)	3.199	0.074
Yes	71 (6.940)	23 (10.455)		
AF, *n* (%)				
No	553 (54.057)	99 (45.000)	5.955	0.015
Yes	470 (45.943)	121 (55.000)		
*β*-receptor blocker using, *n* (%)				
No	332 (32.454)	83 (37.727)	2.264	0.132
Yes	691 (67.546)	137 (62.273)		
ACE-I/ARB, *n* (%)				
No	857 (83.773)	195 (88.636)	3.293	0.07
Yes	166 (16.227)	25 (11.364)		
Calcium inhibitor, *n* (%)				
No	745 (72.825)	192 (87.273)	20.366	<0.001
Yes	278 (27.175)	28 (12.727)		
Diuretic, *n* (%)				
No	240 (23.460)	50(22.727)	0.054	0.816
Yes	783(76.540)	170(77.273)		

MI, myocardial infarction; DM, diabetes; ACE-I/ARB, angiotensin-converting enzyme inhibitor/angiotensin receptor blocker; AF, atrial fibrillation.

### 3.2 Relationship between the RAR and in-hospital mortality and its clinical value

**Figure 1:**
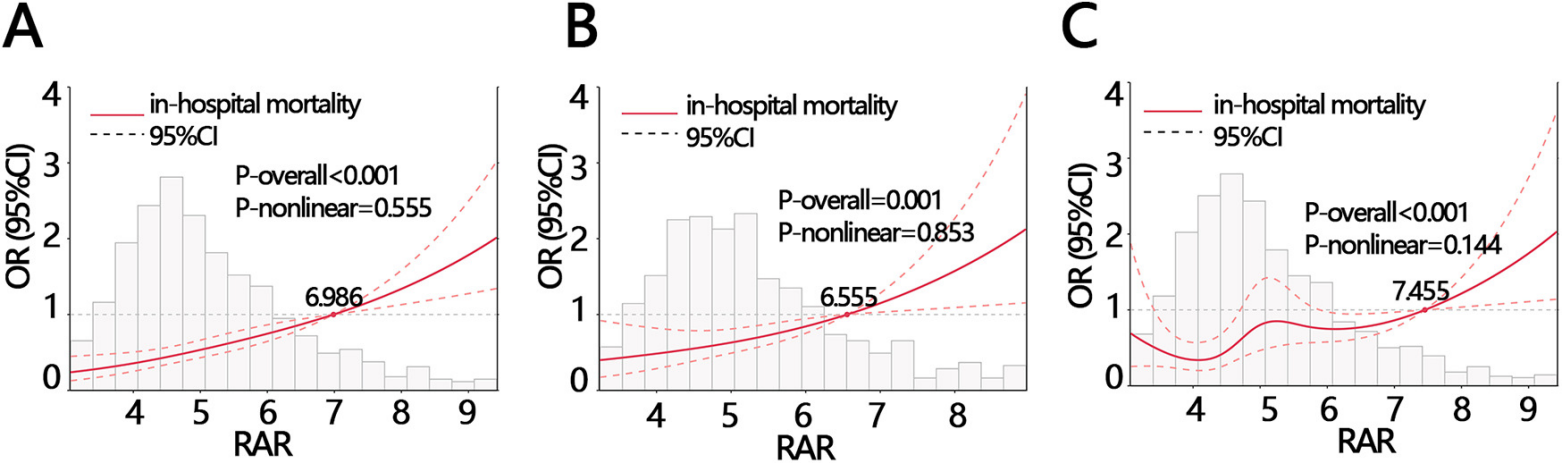
The RCS analysis between RAR and in-hospital mortality with different adjustments **(A)** crude model: without adjustment, **(B)** model 1: adjusting for ALT, anion gap, AST, bicarbonate, calcium, glucose, INR, platelets, total bilirubin WBC, CPK, monocyte, EGFR, **(C)** model 2: adjusting for sex, calcium inhibitor, race, smoking, stroke, AF. ALT, alanine aminotransferase; AST, aspartate aminotransferase; INR, international normalized ratio; WBC, white blood cell; CPK, creatine phosphorylation kinase; EGFR, estimated glomerular filtration rate; RAR, red blood cell distribution width-to-albumin ratio; AF, atrial fibrillation.

**Figure 2:**
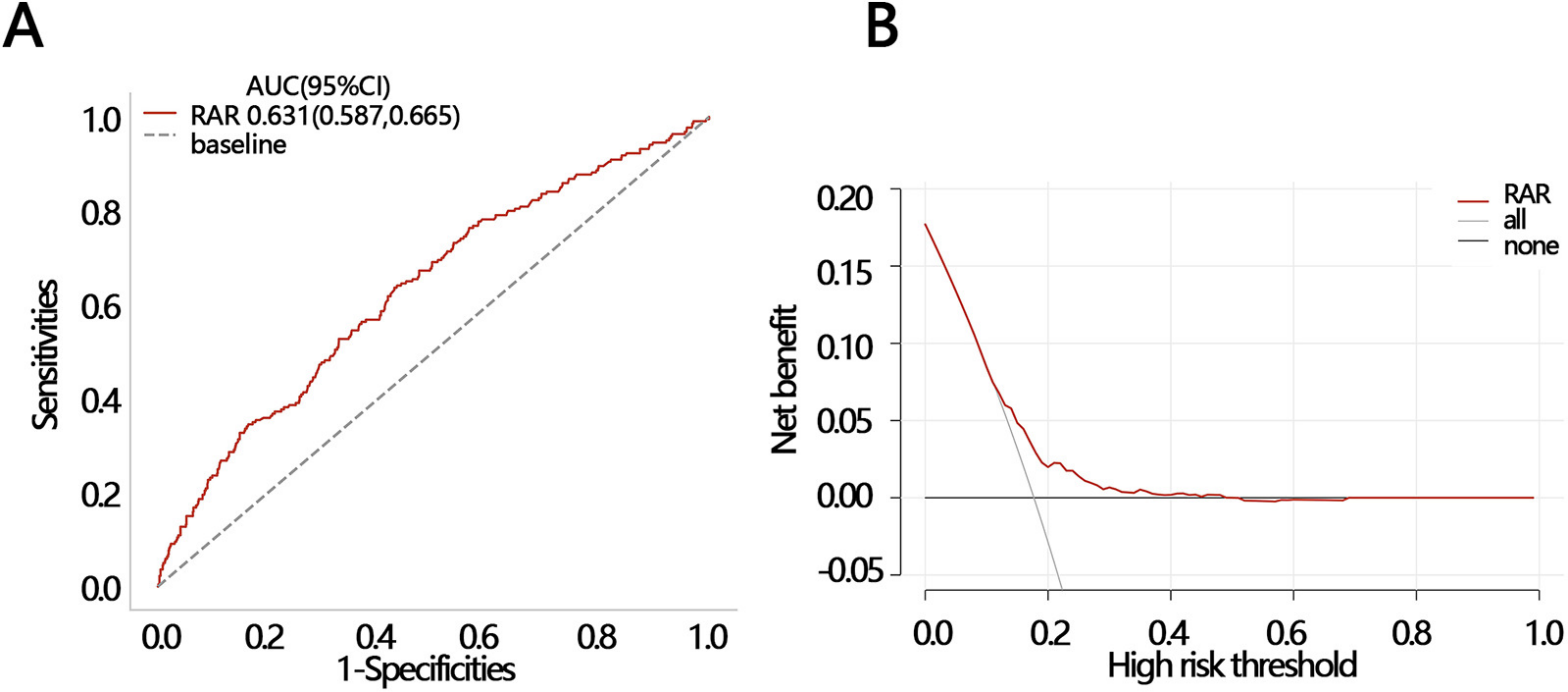
Two methods were used to evaluate the efficacy of RAR in predicting in-hospital death. **(A)** ROC and **(B)** DCA. ROC, receiver operating characteristic curve; DCA, decision curve analysis; AUC, areas under curve; CI, confidence interval.

Logistic regression analysis showed that RAR was a risk factor of in-hospital death (Table 3, OR = 1.388, *P* < 0.05). After controlling for confounders, it was independenlty related to the in-hospital death in both model 1 and model 2 (Table 3, model 1 OR [95% CI]: 1.344[1.145,1.579], model 2, OR [95% CI]: 1.28[1.134,1.445], *P* < 0.05). Logistic regression analysis showed that when RAR was set as a categorical variable according to RAR quartiles, the OR increased with the RAR increasing. The increased trend was not affected by confounders and still held in adjusted model 1 and model 2 (Table 3, *P* for tend <0.05). Therefore, the relationship was further explored using RCS analysis, which showed a linear relationship between RAR and in-hospital deaths and was not affected by confounding factors (crude model: *P*-overall < 0.001, P-nonlinear = 0.555; model 1: P-overall = 0.001, P-nonlinear = 0.853; model 2: P-overall < 0.001, P-nonlinear = 0.144, Figures 1A–C). The results of ROC analysis showed that the AUC value of RAR in predicting patients' in-hospital mortality was: 0.631 [95% CI (0.587,0.665)], and the sensitivity was 0.636 [95% CI (0.358–0.798)], with an accuracy of 0.822 [95% CI (0.801–0.838)] (Figure 2A). The DCA results suggested a net benefit of RAR in predicting patient in-hospital deaths over a threshold probability range of 0.18–0.40 (Figure 2B). The above results suggested that RAR is a risk factor for in-hospital death and has certain value in predicting in-hospital death in patients, which is of significant clinical value.

**Table 3:** Logistic regression analysis investigating the relationship between RAR/quartiles of RAR and in-hospital mortality.

Model	Crude model		Model 1		Model 2	
Variables	OR (95% CI)	*P*	OR (95% CI)	*P*	OR (95% CI)	*P*
RAR	1.388 (1.256, 1.534)	<0.001	1.344 (1.145, 1.579)	<0.001	1.28 (1.134, 1.445)	<0.001
Q1	ref	ref	ref	ref	ref	ref
Q2	1.342 (0.827, 2.177)	0.233	1.279 (0.658, 2.487)	0.469	1.222 (0.731, 2.041)	0.445
Q3	2.049 (1.298, 3.235)	0.002	1.202 (0.635, 2.275)	0.572	1.576 (0.965, 2.575)	0.069
Q4	3.018 (1.944, 4.685)	<0.001	2.533 (1.320, 4.861)	0.005	1.855 (1.147, 3.000)	0.012
*p* for trend		0.014		0.022		0.034

Significant laboratory indices (alive vs. death) from Table 1 and significant clinical features (alive vs. death) from Table 2 were used as adjusted covariates in Model 1 and Model 2, respectively.

Model 1 adjusted for ALT, anion gap, AST, bicarbonate, calcium, glucose, INR, platelets, total bilirubin WBC, CPK, monocyte, EGFR.

Model 2 adjusted for sex, calcium inhibitor, race, smoking, stroke, AF.

RAR, red blood cell distribution width-to-albumin ratio; OR, odd ratio; CI, confidence interval; ALT, alanine aminotransferase; AST, aspartate aminotransferase; INR, international normalized ratio; CPK, creatine phosphorylation kinase; EGFR, estimated glomerular filtration rate; AF, atrial fibrillation.

### 3.3 The mediating role of clinical features significantly associated with RAR

**Figure 3:**
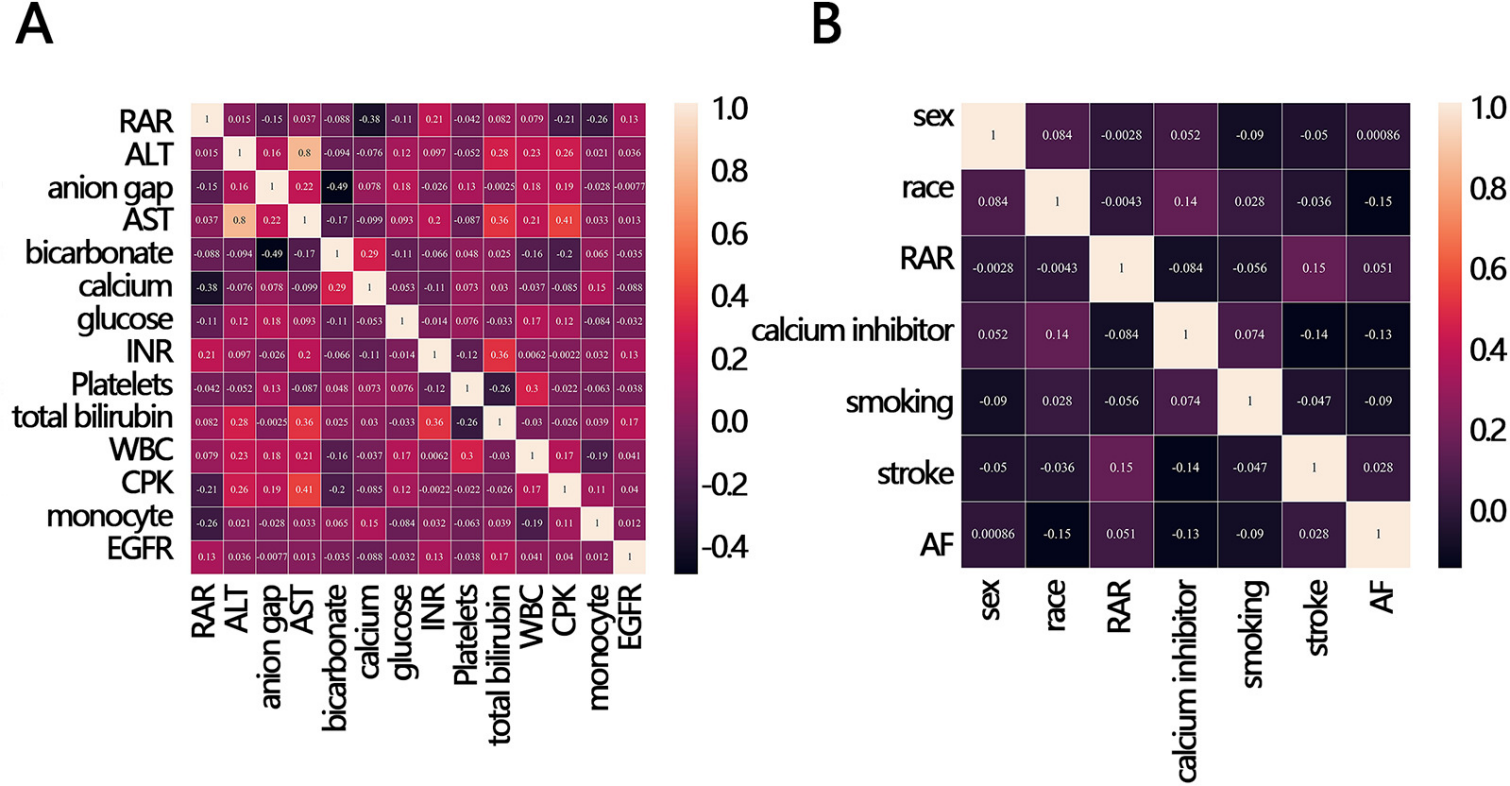
Correlation heat map between RAR and in-hospital mortality-related clinical variables. **(A)** continuous variable, **(B)** categorical variables. ALT, alanine aminotransferase; AST, aspartate aminotransferase; INR, international normalized ratio; WBC, white blood cell; CPK, creatine phosphorylation kinase; EGFR, estimated glomerular filtration rate; RAR, red blood cell distribution width-to-albumin ratio; AF, atrial fibrillation.

**Figure 4:**
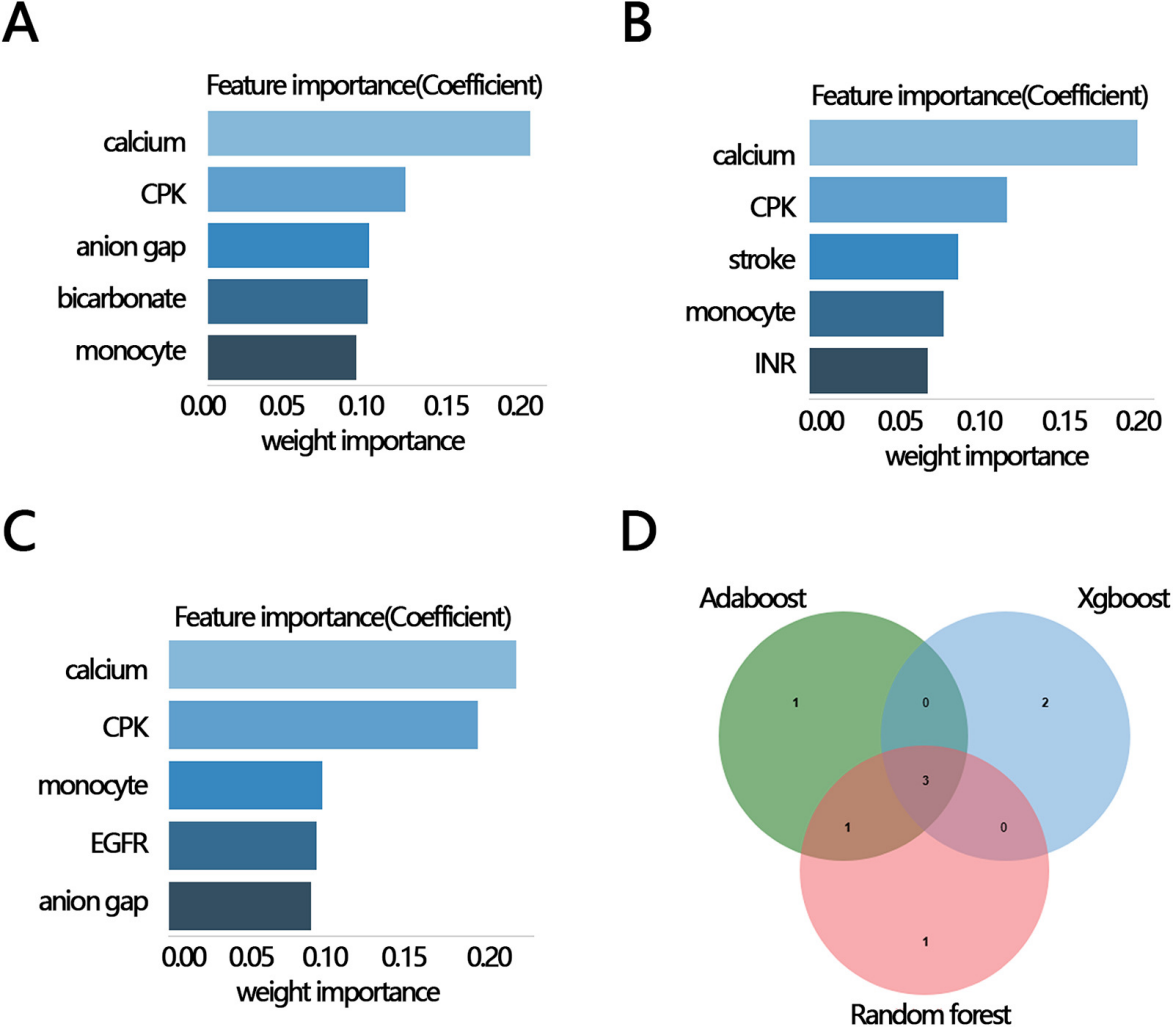
The importance ranking bar chart of the top five RAR-related factors via three methods. **(A)** AdaBoost, **(B)** Xgboost, **(C)** Random forest, **(D)** Venn plot of three kinds of rank variables. INR, international normalized ratio; CPK, creatine phosphorylation kinase; EGFR, estimated glomerular filtration rate; RAR, red blood cell distribution width-to-albumin ratio; AF, atrial fibrillation.

To explore whether other factors mediated the relationship between RAR and in-hospital mortality, we obtained clinical characteristics associated with RAR based on the above indicators that were significantly different between the survival and death groups (except for RAR and RDW) by correlation analysis. The results of the correlation analysis showed that 12 variables, including calcium, CPK, monocyte, EGFR, anion gap, glucose, bicarbonate, INR, stroke, AF, smoking, and calcium inhibitor, were significantly correlated with RAR (Figures 3A,B). These 12 variables were then ranked for importance by three machine learning methods, and top five variables were found including calcium, CPK, anion gap, bicarbonate, monocyte (Figure 4A) for ababoost, a top five ranking of calcium, CPK, stroke, monocyte, INR (Figure 4B) obtained by the Xgboost algorithm, and a top five ranking of calcium, CPK, monocyte, EGFR, anion gap (Figure 4C) obtained by the Random Forest algorithm. The results of Venn showed that calcium, CPK, and monocyte were present in all three top five rankings (Figure 4D), indicating that these three variables were the most relevant clinical characteristics to RAR. Then mediation analysis was performed, and the results indicated that among the three variables, only monocytes mediated the relationship between RAR and in-hospital death (Table 4).

**Table 4:** Mediation analysis among the RAR, RAR-related key factors, and in-hospital mortality.

Variables	Coef (95% CI)	SE	*P*
Calcium			
Calcium∼RAR	−0.221 (−0.259, −0.184)	0.019	<0.001
In-hospital mortality ∼ calcium	−0.035 (−0.057, −0.012)	0.011	0.003
Total	0.053 (0.038, 0.069)	0.008	<0.001
Direct	0.051 (0.034, 0.067)	0.008	<0.001
Indirect	0.003 (−0.002, 0.008)	0.003	0.308
Monocyte			
Monocyte ∼ RAR	−0.332 (−0.476, −0.187)	0.074	<0.001
In-hospital mortality ∼ monocyte	−0.020 (−0.029, −0.010)	0.005	<0.001
Total	0.055 (0.035, 0.076)	0.010	<0.001
Direct	0.050 (0.030, 0.070)	0.010	<0.001
Indirect	0.005 (0.002, 0.011)	0.002	<0.001
CPK			
CPK∼RAR	−14.873 (−59.723, 29.978)	22.85	0.515
In-hospital mortality ∼ CPK	0.000 (0.000, 0.000)	0.000	0.380
Total	0.056 (0.035, 0.077)	0.011	<0.001
Direct	0.056 (0.035, 0.077)	0.011	<0.001
Indirect	0.000 (−0.003, 0.000)	0.001	0.464

CPK, creatine phosphorylation kinase; RAR, red blood cell distribution width-to-albumin ratio; CI, confidence interval; SE, standard error.

### 3.4 The clinical value of RAR in secondary outcomes

**Figure 5:**
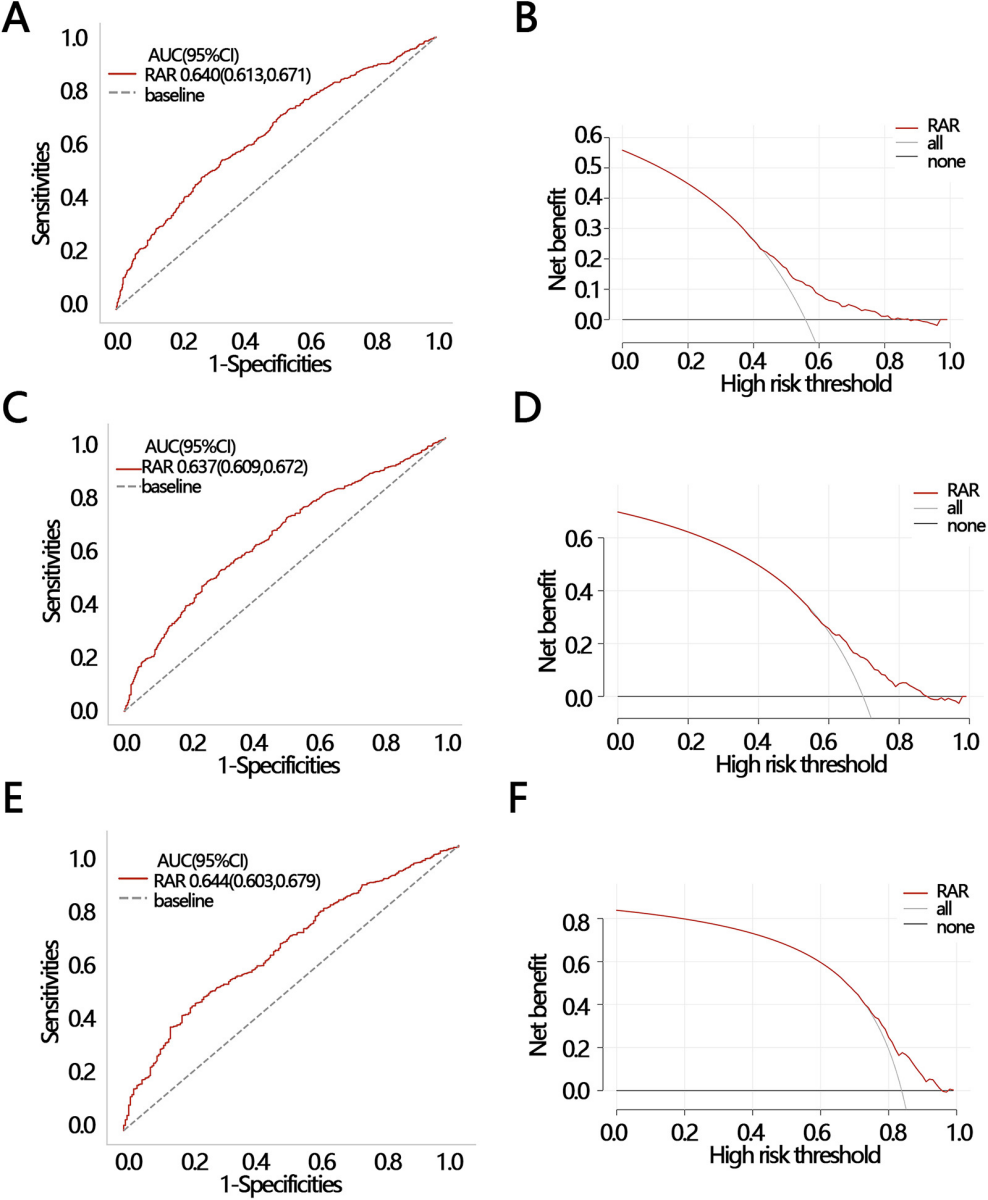
2 ROC and DCA were used to evaluate the clinical value of RAR in different outcomes. **(A)** ROC and **(B)** DCA for half-year mortality; **(C)** ROC and **(D)** DCA for one-year mortality; **(E)** ROC and **(F)** DCA for three-year mortality. ROC, receiver operating characteristic curve; DCA, decision curve analysis; AUC, areas under curve; CI, confidence interval.

To explore the clinical value of RAR in predicting death at follow-up, we analyzed the efficacy and net clinical benefit of RAR in predicting patients' half-year, one-year, and three-year mortality using ROC and DCA. The ROC results showed that the AUC of RAR in predicting patients' death within half-year, one-year, and three-year periods were: 0.640 [95% CI (0.613–0.671)], 0.637 [95% CI (0.609–0.672)], and 0.644 [95% CI (0.603–0.679)], respectively (Figures 5A–E). The sensitivities of the ROC analyses for the three secondary outcomes were 0.549 [95% CI (0.456–0.750)], 0.474 [95% CI (0.434–0.774)], and 0.446 [95% CI (0.369–0.783)]; and the accuracies of the ROC analyses for the three secondary outcomes were 0.444 [95% CI (0.424–0.472)], 0.302 [95% CI (0.283–0.323)], and 0.163 [95% CI (0.141–0.183)], respectively. The DCA results indicated that at a threshold probability of 0.42–0.81, RAR was of net benefit in predicting patient death within half-year (Figure 5B); at a threshold probability of 0.63–0.92, RAR was of net benefit in predicting one-year survival of patient (Figure 5D); at a threshold probability of threshold probabilities of 0.79–0.10, RAR had a net benefit in predicting three-year survival of patient (Figure 5F). These results suggest that RAR has significant value in predicting patient death at follow-up.

## 4 Discussion

The pathogenesis of CHF combined with CKD is a complex condition, involving multiple mechanisms such as CKD resulting from heart failure, inflammatory responses, and neurohormonal activation (15, 16). RAR is a novel composite marker reflecting the body's immune and nutritional status (17). In this study, we found that RAR was significantly associated with the risk of in-hospital mortality, and this relationship was linear among patients with CHF combined with CKD. Furthermore, we also demonstrated that RAR serves as a strong predictor of patient survival, with good prognostic value for predicting 6-month, 1-year, and 3-year survival.

It had been suggested that RAR is associated with poor prognosis in various diseases, including coronary artery disease (10), heart failure (11), sepsis (13), aortic aneurysm (14), cancer (18), and autoimmune encephalitis (19). In our study, we also found that RAR was an independent risk factor of in-hospital mortality in patients. This may be related to inflammation and oxidative stress. In the context of CHF combined with CKD, CKD induces both local and systemic inflammatory responses. Cytokines such as tumor necrosis factor-alpha (TNF-α) and interleukin-6 (IL-6) are elevated, while oxidative stress can damage endothelial cells, increase vascular permeability, and further contribute to sodium and water retention and tubular damage. The interaction between induced oxidative stress and inflammation exacerbates the functional impairment of both the heart and kidneys (20). Furthermore, inflammation suppresses iron metabolism and erythropoietin production, leading to the release of more immature cells into the bloodstream, which results in an increase in RDW (21, 22). Additionally, studies have shown that inflammatory cytokines inhibit red blood cell maturation and promote the entry of immature red blood cells into peripheral circulation, thereby causing an increase in RDW (22).

Albumin is also a biomarker of inflammation and nutritional status. Inflammatory processes lower albumin levels, primarily due to the elevated expression of cytokines such as IL-6 and nitric oxide (NO), which induce the level of vascular endothelial growth factor (VEGF) to rise. This leads to increased microvascular permeability and angiogenesis, altering the distribution of albumin between the intravascular and extravascular spaces, thus lowering serum albumin concentration (23, 24). Moreover, most studies suggest that a decrease in RAR is associated with poor prognosis in various diseases (25). In summary, during inflammatory states, RAR is elevated, reflecting the degree of inflammation and is associated with poor patient prognosis. It is noteworthy that patients with concomitant CHF and CKD commonly present advanced age, volume overload (26, 27), and hepatic congestion. These characteristics collectively influence RAR levels and adversely impact prognosis. Advanced age is frequently associated with immunosenescence and impaired nutrient absorption (28), which may directly or indirectly elevate RAR. Volume overload potentially induces hemodilution and altered vascular permeability, both of which reduce serum albumin concentration and consequently increase RAR (29). Impaired cardiac function leads to systemic venous congestion, including compromised hepatic venous drainage, resulting in hepatic congestion [Liver abnormalities in heart failure]. Hepatic sinusoidal distention compresses hepatocytes, causing hepatocellular atrophy and mitochondrial dysfunction. This reduces ATP synthesis and impairs albumin production (30), thereby elevating RAR. Thus, the clinical significance of elevated RAR may lie in its integrative reflection of these intertwined prognostic risk factors, particularly the combination of inflammation and malnutrition.

Monocytes are key mediators of innate immunity (31). In this study, we also found that monocytes mediate the relationship between the RAR and in-hospital mortality. Patients with CHF often exhibit a systemic inflammatory state, with monocytes playing a central role in this process. By secreting cytokines such as TNF-α, interleukin-1 (IL-1), and IL-6, as well as chemokines, monocytes contribute to both local and systemic inflammation, leading to structural and functional damage to the heart and kidneys (32, 33). In the context of CHF combined with kidney disease, monocytes may exacerbate the interactions between the heart and kidney, affecting prognosis (34). RAR reflects the inflammatory and immune status within the patient's body. A higher RAR indicates a more severe stress response, with further activation of monocytes through their pro-inflammatory actions. This amplifies the deterioration of both heart and kidney functions, ultimately increasing the risk of in-hospital mortality.

Currently, several researchers have been focusing on the prediction of in-hospital mortality in patients with CHF and CKD. For example, Li et al. established a XGBoost model using 22 factors, including sequential organ failure assessment (SOFA) score, age, simplified acute physiology score II (SAPS II), and urine output, and achieved a prediction performance with AUC = 0.587 (35). The XGBoost model validated the potential of multi-metric federation. Chen et al. employed 13 variables and constructed a prognostic model, and the AUC of the model was 0.771 (36), which demonstrated the value of multidimensional assessment in specific populations. The AUC value of RAR identified in our study for predicting in-hospital mortality was 0.631, which was moderate. The core difference between our study and existing research is that, moderate predictive power is achieved with a minimalist metric (RDW/ALB ratio), while taking into account clinical operability and biological rationality. Compared with other studies, our research focused on the useful clinical routine indicators, and was designed to provide more practical tools for scenarios with limited resources or where rapid assessment is required. Furthermore, RAR demonstrates predictive capability not only for in-hospital mortality but also for half-year, one-year, and three-year mortality.

In other factors, our study has some strengths. First, this study is the first to reveal the relationship between the RAR and in-hospital mortality in patients with CHF combined with CKD Our study suggested the index simplicity and clinical operability. Only two routine laboratory indicators are required, eliminating the need for complex or expensive tests (e.g., genetic testing, dynamic imaging), significantly reducing the difficulty of data collection and the cost of clinical application. Second, RAR reflects the integration of multidimensional pathological information. RDW and albumin reflect different pathophysiological states, respectively, and their ratios may capture the core drivers of disease deterioration, while the multi-indicator model may mask key signals due to redundancy or correlation of indicators. Third, the black box nature of multi-metric models (such as XGBoost's complex algorithm) often makes it difficult for clinicians to understand the prediction logic, limiting its practical application. The RDW/ALB ratio is highly interpretable.

However, this study has certain limitations. First, the data used in this study were derived from the MIMIC database, which may have limitations related to racial diversity. Second, the study lacked measurements of RAR at multiple time points due to excessive missing data, preventing further exploration of the association between dynamic changes in RAR and mortality outcomes. Third, this study had several limitations regarding data availability. Important clinical indicators of heart failure severity-including NYHA functional classification, left ventricular function/dimensions, arrhythmia documentation, and B-type natriuretic peptide were not available in the MIMIC-IV database. C-reactive protein, NT-proBNP, and hs-C-reactive protein were excluded from analysis due to excessive missing data, rendering them unsuitable for meaningful statistical evaluation. Neutrophils, lymphocytes, and other immune-related indicators were also not available, leading to the inability to further explore the impact of immunity on the prognosis of patients.

## 5 Conclusion

This study released the positive linear relationship between RAR and in-hospital mortality, which provided a new biomarker for predicting the prognosis in patients with CHF combined with CKD. The study found that monocytes played a mediating role in the relationship between RAR and in-hospital mortality. In addition, the RAR had a good function for predicting the 6-month, 1-year, and 3-year survival.

## Data Availability

The raw data supporting the conclusions of this article will be made available by the authors, without undue reservation.
