# Supplementary material for: Association between red blood cell distribution width-to-albumin ratio and in-hospital mortality in patients with congestive heart failure combined with chronic kidney disease
Source: Front Cardiovasc Med. 2025 Jul 4;12:1563512. doi: 10.3389/fcvm.2025.1563512 (PMC12271087; doi:10.3389/fcvm.2025.1563512)
Supplement: Supplementary file 1 [file Datasheet1.docx]

Supplementary Document 1

**ICD-code of CHF:** 4280, 39891, 42823, 15020, 15021, 15022, 15023, 15030, 15031, 15032, 15033, 15041, 15043.

**ICD-code of CKD:** 28521, 40300, 40301, 40310, 40311, 40390, 40391, 40400, 40401, 40402, 40403, 40410, 40411, 40412, 40413, 40490, 40491, 40492, 40493, 5851,5852,5853, 5854, 5855, 5859, D631, E0822, E0922, E1022, E1122, E1322, I12, I120, I129, I13, I130, I131, I1310, I1311, I132, N18, N181, N182, N183, N184, N185, N189, O102, O1021, O10211, O10212, O10213, O10219, O1022, O1023, O103, O1031, O10311, O10312, O10313, O10319, O1032, O1033.
